# Editorial: Unravelling the unknown of the rumen microbiome: implications for animal health, productivity, and beyond

**DOI:** 10.3389/fmicb.2025.1720795

**Published:** 2025-10-31

**Authors:** Alejandro Belanche, Grzegorz Belzecki, Emma Hernandez-Sanabria, Gonzalo Martínez-Fernandez, Eva Ramos-Morales, Gabriel de la Fuente

**Affiliations:** ^1^Departamento de Producción Animal y Ciencia de los Alimentos, Universidad de Zaragoza, Zaragoza, Spain; ^2^Department of Animal Nutrition, The Kielanowski Institute of Animal Physiology and Nutrition, Polish Academy of Sciences, Jabłonna, Poland; ^3^Trouw Nutrition Innovation R&D, Boxmeer, Netherlands; ^4^Agriculture and Food, CSIRO, St Lucia, QLD, Australia; ^5^Estación Experimental del Zaidín (CSIC), Granada, Spain; ^6^Departament de Ciència Animal, Universitat de Lleida – Agrotecnio Center, Lleida, Spain

**Keywords:** early-life interventions, feed additives, feed efficiency, methanogenesis, rumenmicrobiome

The rumen, a highly specialised fermentation chamber within the digestive systems of ruminant animals, harbours a complex and dynamic microbial ecosystem. This intricate consortium of bacteria, archaea, protozoa, and fungi plays a central role in the degradation and fermentation of fibrous plant material, ultimately converting it into absorbable nutrients and energy for the host. Beyond its fundamental role in nutrient metabolism, the rumen microbiota exerts profound effects on animal health, immunity, productivity, and environmental sustainability. Understanding its structure and function is therefore critical not only for improving livestock production but also for advancing sustainable agricultural systems in the face of global challenges such as climate change and food security.

Despite major progress in ruminant nutrition and microbiology, many aspects of the composition, functional diversity, and ecological dynamics of the rumen microbiota remain insufficiently characterised. Recent advances in high-throughput multi-omics approaches have revolutionised our ability to dissect microbial functions and interactions, paving the way for mechanistic insights into host–microbe symbiosis. This Research Topic was designed to consolidate cutting-edge contributions that bridge existing knowledge gaps, providing a deeper understanding of rumen microbial ecology and its implications for host physiology, productivity, and environmental outcomes.

The contributions to this Research Topic demonstrate the value of a multidisciplinary approach that combines omics-driven exploration of microbial communities with applied nutritional sciences and systems biology. The articles tested novel hypotheses and offered practical solutions; moreover, they provided a comprehensive and updated overview of the rumen microbiome and its complex interplay with ruminant physiology, nutrition, and health. A total of 45 manuscripts were submitted, of which 29 (including one corrigendum) were accepted. These cover a wide range of ruminant species (cattle, sheep, goats, yaks, and red deer) along with *in vitro* studies. The published articles can be classified into the following categories.

## Novel feeds and agro-industrial by-products

Eight articles explored the use of agro-industrial by-products and novel feeds in ruminant nutrition, including a review of the degradation mechanisms of lignocellulose by ruminants. Li, Xu, et al. studied the impact of chilli straw supplementation in sheep and found improvements in growth performance and beneficial changes in fungal populations at a 10% inclusion rate. A companion publication demonstrated that rumen fermentation was also enhanced when this by-product was included at different proportions in the diet (Li, Tuo, et al.). Hou G. et al. evaluated black goji berry (*Lycium ruthenicum*) as forage at a level of 20%−30% inclusion, reporting improved growth performance and overall health in sheep, with increased apparent NDF digestibility and immune system indicators, without compromising liver function. The use of saline pasture in Qinghai Tibetan sheep was investigated by Jia et al.. A mixture of salt-alkali-tolerant grasses was compared with common non-saline grass pastures in 60 Tibetan rams (2–3 months old). The results showed significant changes in meat quality traits, such as colour, in addition to increased amino acid content. Gene expression related to protein metabolism was upregulated, and higher concentrations of antioxidants, such as flavonoids, were detected.

Three studies investigated the use of by-products in Hu sheep. Ren et al. reported that oat grain supplementation induced significant shifts in microbial composition, increasing the abundance of Acidobacteriota, Proteobacteria, Chloroflexi, and Actinobacteriota, in addition to overall alpha diversity. Metabolically, oat grain supplementation reduced isobutyric acid and citraconic acid while increasing azelaic acid. Jiao et al. tested three corn varieties in whole-plant silage and found that two of them improved lamb production by increasing dry matter intake, growth performance, and gastrointestinal fermentation. These findings highlight the importance of selecting appropriate corn silage varieties to optimise both production performance and animal health, offering novel insights into the interplay between dietary composition and the rumen microbiota. Lu et al. evaluated spent mushroom substrate (*Pleurotus ostreatus*), a by-product consisting of residual plant biomass after mushroom cultivation. Inclusion levels of 5%, 10%, 15%, and 20% were tested in Hu sheep diets. High doses were detrimental to feed utilisation efficiency, blood oxygen-carrying capacity, kidney function, growth performance, and rumen function. However, moderate inclusion (≤ 10%) enhanced rumen absorption capacity.

Finally, Fu et al. presented a review focusing on lignocellulose degradation in the rumen. The authors discussed the enzymatic mechanisms of fibre digestion, the interactions among microbial populations, and the role of currently uncultured microorganisms. They highlighted research priorities such as developing new culture techniques, integrating molecular biology and biochemistry, and improving enzyme efficiency through genetic engineering and other biotechnological approaches.

## Optimisation of the rumen microbial fermentation

Three articles addressed strategies to optimise rumen microbial fermentation. Viquez-Umana et al. studied the effects of protein levels and feeding patterns, showing that the rumen microbiome and fermentation patterns (i.e., pH and VFA proportions) remained stable despite variations in dietary CP supply, suggesting compensatory mechanisms. In yaks, Gou et al. demonstrated that optimising concentrate-to-forage ratios is critical to balancing production goals, rumen pH, and feed costs. Bai et al. highlighted microbial shifts that support adaptation to cold-season environments, including enhanced fibre degradation, total VFA production, higher acetate proportions, and reduced nitrogen excretion.

## Use of bioactive compounds

Six research articles investigated the effects of bioactive compounds and feed additives on rumen microbiota, productivity, and health. Klein et al. evaluated tannin supplementation in dairy cattle by analysing the faecal microbiome and associated metabolites. Their results showed that tannins can modulate gut microbial ecology with minimal effects on manure-related emissions. Xiao et al. tested quercetin (a flavonoid widely distributed in plants) *in vitro* and observed reduced methane production and improved nitrogen utilisation. While archaeal diversity remained unaffected, the abundance of specific genera, such as *Methanobrevibacter*, was altered. Wang et al. assessed the impact of steviol glycosides in 24 Holstein calves supplemented for 8 weeks. The authors reported morphological changes in digestive organs and rumen papillae, which led to increased total VFA production. Xie et al. studied the effects of dietary supplementation with zinc oxide nanoparticles in dairy goats, reporting increased milk yield and fat content, and enhanced ruminal alpha diversity. Untargeted metabolomics identified 261 differential metabolites associated with the supplementation. Zhang et al. tested enzyme blends (carbohydrases and peptidases) on yaks and evaluated performance, meat quality, and microbial composition in the subjects studied. Their findings provide insights into optimising feeding management and microbe–host interactions. Finally, Fan et al. investigated the effects of L-citrulline supplementation on rams, showing its beneficial effects on sperm quality alongside rumen microbial composition modulation.

## Inhibition of rumen methanogenesis

Four articles addressed the mitigation of rumen methanogenesis. Martinez-Fernandez et al. demonstrated that supplementation of cattle feed with commercial wood biochar or custom-made wheat straw biochar with nitrates reduced their methane emissions by up to 12.9% under controlled feeding conditions; however, no effect was observed in grazing systems. The authors concluded that effective methane mitigation in grazing animals requires additives with greater efficacy and persistence in the rumen to overcome the variability of grazing environments. Pu et al. reported that replacing corn silage with fermented bakery by-products reduced methane production in beef calves by shifting rumen fermentation from acetate to propionate.

Two reviews provided broader perspectives on methane mitigation strategies. Frazier et al. examined the links between the rumen microbiome and climate change, discussing functional redundancy, microbial succession, and methane mitigation within ecological theory frameworks (e.g., Black Queen Dynamics, Island Biogeography Theory, and Neutral Theory). Pressman and Kebreab critically evaluated mechanistic models of rumen fermentation, focusing on their ability to predict methane emissions based on feed fractions, microbial groups, fermentative products, rumen pH, redox balance, and passage rates. Together, these works provide a comprehensive overview of methane modelling and its application to rumen function.

## Microbial correlations as a predictive tool

Four publications, plus one corrigendum [Skarlupka et al.(a)], proposed the use of gut microbial correlations as a predictive tool. Yang et al. reported correlations between rumen and rectal bacterial composition and dairy cow performance, linking specific microbial communities with increased propionate production, enhanced gluconeogenesis, and reduced inflammation. In addition, Skarlupka et al.(b) developed a novel, noninvasive colourimetric method using oral swabs, finding that swab colouration correlated with microbial composition. Hou L. et al. investigated acid–base imbalances and identified associations between altered rumen pH, microbial composition, and lipopolysaccharide biosynthesis in dairy cows, offering new insights into the diagnosis and prevention of SARA and systemic inflammation. Moreover, Wu et al. studied the effects of yeast-fermented feed supplementation on dairy cows, showing promising effects in reducing the incidence of mastitis under high-concentrate diets.

## Gut microbiota and host genetics

Du et al. investigated the influence of host genetics by comparing Hu, East Friesian, and crossbred sheep. Their results showed that crossbreds showed improved energy efficiency and environmental adaptability, potentially due to inherited microbial functions and complementary metabolic traits. Xu et al. further identified microbial taxa (notably Firmicutes and Lachnospiraceae) associated with fiber degradation efficiency in Hu sheep, underscoring the relevance of currently uncultured microbes. Finally, Wei et al. explored hybridisation in red deer (*Cervus elaphus songaricus, Cervus elaphus yarkandensis*, and their hybrids) and demonstrated microbiome and metabolome shifts with potential applications in probiotic development and microbial-assisted breeding for enhanced antler growth and health.

